# Precision of orthodontic cephalometric measurements on ultra low dose-low dose CBCT reconstructed cephalograms

**DOI:** 10.1007/s00784-021-04127-9

**Published:** 2021-08-28

**Authors:** R. H. van Bunningen, P. U. Dijkstra, A. Dieters, W. J. van der Meer, A. M. Kuijpers-Jagtman, Y. Ren

**Affiliations:** 1 Department of Orthodontics, University Medical Center Groningen, University of Groningen, Hanzeplein 1, 9713 GZ Groningen, The Netherlands; 2 Department of Rehabilitation and Department of Oral and Maxillofacial Surgery, University Medical Center Groningen, University of Groningen, Hanzeplein 1, 9713 GZ Groningen, The Netherlands; 3 Department of Orthodontics and Dentofacial Orthopedics, School of Dental Medicine/Medical Faculty, University of Bern, Freiburgstrasse 7, CH-3010 Bern, Switzerland; 4 Faculty of Dentistry, Universitas Indonesia, Campus Salemba, Jalan Salemba Raya No. 4, Jakarta, 10430 Indonesia

**Keywords:** Cephalometry, Cone beam computed tomography, Orthodontics, Diagnosis, Accuracy, Variation, Reliability

## Abstract

**Objectives:**

To analyze differences in variation of orthodontic diagnostic measurements on lateral cephalograms reconstructed from ultra low dose-low dose (ULD-LD) cone beam computed tomography (CBCT) scans (RLC) as compared to variation of measurements on standard lateral cephalograms (SLC), and to determine if it is justifiable to replace a traditional orthodontic image set for an ULD-LD CBCT with a reconstructed lateral cephalogram.

**Material and methods:**

ULD-LD CBCT images and SLCs were made of forty-three dry human skulls. From the ULD-LD CBCT dataset, a lateral cephalogram was reconstructed (RLC). Cephalometric landmarks (13 skeletal and 7 dental) were identified on both SLC and RLC twice in two sessions by two calibrated observers. Thirteen cephalometric variables were calculated. Variations of measurements, expressed as standard deviations of the 4 measurements on SLC and RLC, were analyzed using a paired sample *t*-test. Differences in the number of observations deviating ≥ 2.0 mm or degrees from the grand mean between SLC and RLC were analyzed using a McNemar test.

**Results:**

Mean SDs for 7 out of 13 variables were significantly smaller for SLCs than those for RLCs, but differences were small. For 9 out of 13 variables, there was no significant difference between SLC and RLC for the number of measurements outside the range of 2 mm or degrees.

**Conclusions:**

Based on the lower radiation dose and the small differences in variation in cephalometric measurements on reconstructed LC compared to standard dose LC, ULD-LD CBCT with reconstructed LC should be considered for orthodontic diagnostic purposes.

**Clinical relevance:**

ULD-LD CBCT with reconstructed LC should be considered for orthodontic purposes.

**Supplementary Information:**

The online version contains supplementary material available at 10.1007/s00784-021-04127-9.

## Introduction

For orthodontic diagnosis, treatment planning, treatment progress evaluation, and monitoring of growth and development, traditionally two-dimensional panoramic and lateral cephalograms (LC) are indispensable tools. Limitations of two-dimensional radiographs are magnification, distortion, and over-projection of anatomical structures. Panoramic radiographs (PAN) and LCs provide 2-dimensional information about osseous, dental, and soft tissue relationships, but not about three-dimensional, unilateral, or transverse aspects of the craniofacial complex. An additional third dimension may enhance orthodontic diagnosis and treatment planning [1, 2].

Until recently, only in selected cases, the need for more diagnostic information allowed the use of a small field of view cone beam computed tomography scan (CBCT) because it adds to the total radiological dose [1–7]. Several studies compared the effective doses of different digital radiographic methods with CBCT measured for different devices. The effective dose of the CBCT was between 5 and 7 times higher than the combined doses of a PAN and LC. The overall effective dose of a standard dose PAN plus LC was 26.9 μSv (PAN 21.87 μSv + LC 5.03 μSv) [8] or 30 μSv (PAN 27.1 μSv + LC 2.50 μSv) [9] versus an overall effective dose of a CBCT of 132 μSv [8] or 210 μSv [9]. The doses mentioned in the research of Signorelli (2016) [8] and Chinem (2016) [9] were measured on different machines (e.g., Signorelli: KaVo 3D eXam, Chinem: Heliodent Plus (Sirona Dental Systems, Bensheim, Germany), Orthophos XG 5 (Sirona Dental Systems, Bensheim, Germany), and i-CAT (Imaging Sciences International, Hatfield, PA, USA).

Studies comparing cephalometric measurements performed on a conventional LC with those on a CBCT-reconstructed LC found no significant differences in measurements on CBCT reconstructed cephalograms and those based on conventional radiographic images. In these studies, CBCT images were made using standard dose settings [10–13]. It was concluded that CBCT-reconstructed LCs can successfully replace SLCs.

Since then, ultra low dose (ULD) and ultra low dose-low dose (ULD-LD) CBCT protocols have become available. These ULD-LD protocols provide an 87% reduction in dose compared with the standard exposure protocols in both child and adult phantoms [14, 15]. From these datasets, a LC can be reconstructed (RLC). The effective dose of an ULD-LD CBCT ranges from 11 μSv for an adult to 18 μSv for a child [14, 15, 17]. The doses mentioned in the research of Ludlow (2013) [14] were measured using an i-CAT FLEX Next Generation dental CBCT unit (Imaging Sciences) using Quickscan plus settings. The doses mentioned in the research of Ludlow (2015) [17] were measured on the same machine we used, using ULD-LD settings. All measurements were done using phantom heads with dosimeters.

Because of their three-dimensional nature, CBCTs contain more information with less over-projection than a single PAN, so visibility of structures is better on a CBCT than on a conventional PAN [2]. Until today, due to the lack of three-dimensional cephalometric reference values for orthodontic diagnosis and treatment planning, a two-dimensional cephalometric analysis is the most common, which can be reconstructed from the ULD-LD CBCT scan.

When differences in variation of measurements on lateral cephalograms reconstructed from ULD-LD CBCT scans and on standard dose LCs are small, a single ULD-LD CBCT could become the standard in orthodontics. Especially as the latter image modality provides additional three-dimensional information and contributes to a radiological dose reduction.

The aims of this study were to analyze differences in variation of orthodontic diagnostic measurements on lateral cephalograms reconstructed from ULD-LD CBCT scans (RLC) as compared to the variation of measurements on standard lateral cephalograms (SLC), and to determine if it is justifiable to replace a traditional orthodontic image set for an ULD-LD-CBCT with a reconstructed lateral cephalogram.

## Materials and methods

### Skulls

Forty-three dry human skulls were selected from an existing collection at the Department of Orthodontics at the University Medical Center Groningen (UMCG), the Netherlands. The selection of the skulls was based on the development of the dentition. All skulls were at least at the end of the first transitional phase, so all permanent anterior teeth and first molars had erupted. The Institutional Medical Ethics Review Board judged that no ethical approval is required (#METc: 2019/616).

### Preparation of the skulls

**Fig. 1:**
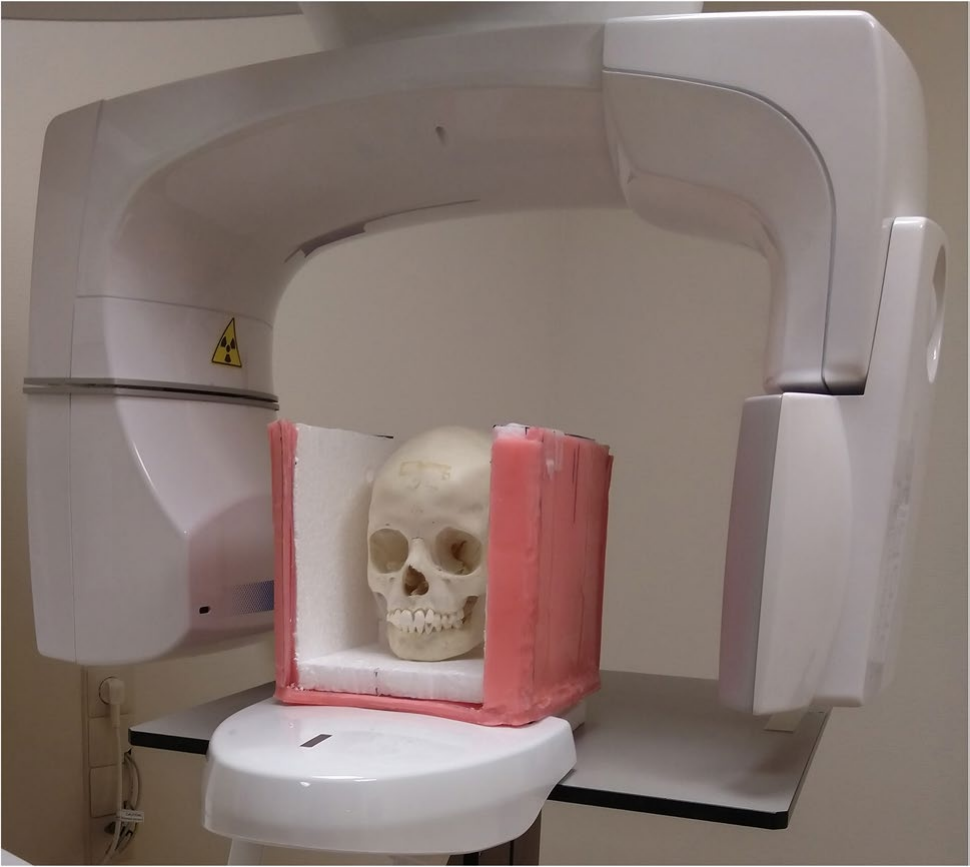
Dry skull in EPS box with 1-cm utility wax positioned in CBCT machine (front part removed for photo)

For each skull, the mandible was anatomically positioned to the maxilla with the condyle in the fossa and all teeth in a stable occlusion using 3 M Scotch tape (3 M Saint Paul, MN, USA) fixing the mandibular ramus to the temporal bone on both sides of the skull. Then, the skulls were placed on expanded polystyrene (EPS) blocks in natural head position. To simulate soft tissues, the skulls were placed in an EPS box with 2-cm-thick walls to which a 1-cm-thick layer of utility wax (Fig. 1) was applied. This material is effective in simulating soft tissue in most regions [16].

### Radiographs

The skulls were scanned using a Planmeca ProMax 3D Mid (Planmeca Oy, Helsinki, Finland). Each skull was positioned in the box as described above, and put in the center of the CBCT scanner, using laser positioning beams to coincide with the midsagittal plane.

**Fig. 2:**
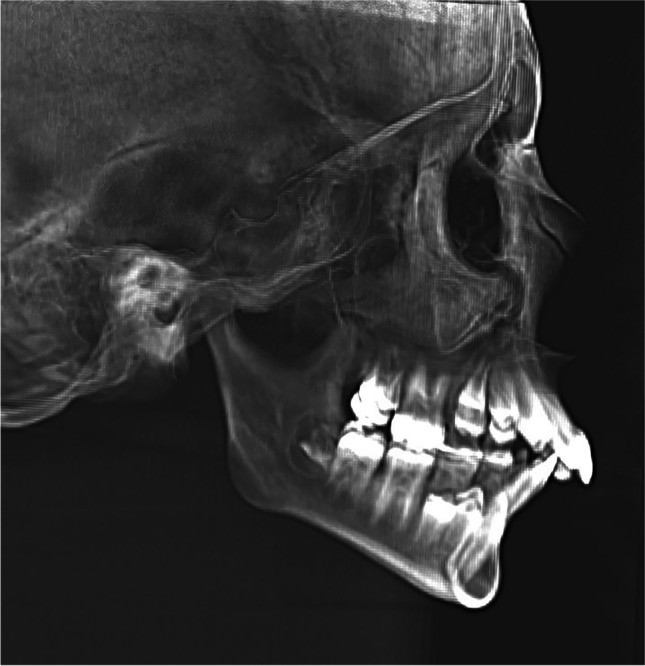
Reconstructed lateral cephalogram from ultra low dose-low dose CBCT

First ultra low dose-low dose computerized tomography scans (ULD LD CBCT) were made using a 600-mm voxel size scan with a diameter of 20.0 cm and height of 17.5 cm at 2.2 mA and 90 kV for 9 s. The effective dose per skull was 18 μSv (Planmeca Oy, Helsinki, Finland), as it was measured by Ludlow et al. (2015) using the same equipment and settings [17]. The effective dose was also calculated by our clinical physicist using a Monte Carlo simulation. The total effective dose was calculated at 16 µSv. From the ULD-LD CBCT dataset, a lateral cephalogram was reconstructed (RLC) using ROMEXIS software (Fig. 2).

**Fig. 3:**
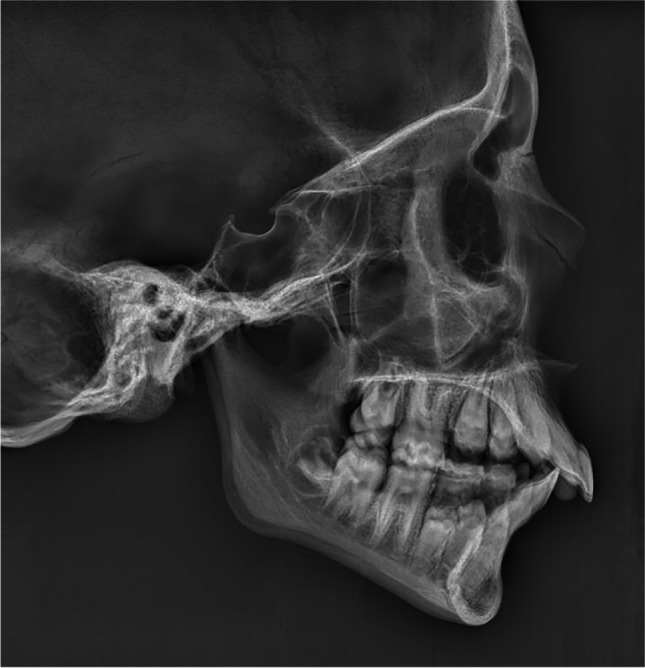
Conventional lateral cephalogram

After the ULD LD CBCT was made, the EPS box with skull was moved to the cephalostat of the same machine. The skulls were positioned in natural head position on visual estimation in relation to the vertical measurement nose-rod. Standard dose lateral cephalometric radiographs (SLC) were taken at 10 mA and 66 kV for 6.79 s (Fig. 3). These exposure factors are the standard factory protocol adult settings for a normal dose LC. The effective dose was calculated using these settings using a Monte Carlo simulation. The total effective dose was calculated at 1 µSv.

All images were stored in JPEG format and loaded into Viewbox cephalometric tracing software (dHAL Software, Kifissia, Greece). Both SLCs and RLCs were scaled to true dimensions.

### Cephalometry

Cephalometric landmarks (13 skeletal and 7 dental) were identified on both SLC and RLC (Supplementary table 1). For the cephalometric analysis, 10 conventional angles (degrees) and 3 distances (mm) were calculated (Supplementary table 2).

On both SLC and RLC of each skull, the landmarks were identified in 2 sessions on 2 occasions (2 weeks apart) by the same observer (RvB). In the first week (occasion 1), the landmarks were indicated twice (sessions 1 and 2) on 43 SLC and 43 RLC images. The sequence of the images was random. After 2 weeks (occasion 2), the same procedure was repeated, resulting in 8 datasets: 4 for the SLC and 4 for the RLC. A radiodiagnostic technician (AD) performed the same procedure independently on 10 randomly selected skulls.

Both observers were experienced in orthodontic radiodiagnostics and were calibrated before the measurements were performed.

### Statistical analyses

To determine differences in variation, for each skull, 2 standard deviations (SD) were calculated one for the 4 measurements of the SLC and one for the 4 measurements of the RLC for each of the outcome variables. Differences in standard deviations of the SLC and the RLC were analyzed using a paired sample *t*-test. Thereafter, the grand mean per skull was calculated for the 8 measurements per outcome variable. The number of observations with a difference ≥ 2.0 mm or degrees from the grand mean was calculated per skull for each outcome variable and for each type of radiograph. This procedure was followed because prior to our study it was unknown which type of radiograph leads to more accurate measurements. The grand mean is based on all measurements of both types of radiographs. Differences in the number of observations ≥ 2.0 mm or degrees from the grand mean between SLC and RLC were analyzed using a McNemar test. Observations < 2.0 mm or degrees were considered clinically acceptable [12, 18–20].

Intraclass correlation coefficients, single measure, absolute agreement, and two-way random model (ICC) were calculated as a measure for intra-observer reliability and inter-observer reliability of the measurements of observer 1 (RvB) and 2 (AD).

All statistical analyses were performed using IBM SPSS Statistics vs. 23 (SPSS, Chicago, IL).

## Results

### Variation

Standard deviations of the SLC as a measure for variation were significantly smaller for SNA, SNB, ANB, ANS-PNS/GoGn, Occl/SN, SN/GoGn, and Upper inc. / ANS-PNS compared to those of RLC (Table 1).

**Table 1:** Differences in variation, of linear and angular measurements performed on standard lateral cephalograms (*SLC*) and reconstructed lateral cephalograms (*RLC*)

Variable	SLC Variation Mean (sd)	RLC Variation Mean (sd)	Difference in means	95% CI	*p*#
**Skeletal**					
SNA (°)	0.64 (0.63)	0.88 (0.47)	0.25	0.10; 0.39	0.002
SNB (°)	0.65 (0.52)	0.84 (0.36)	0.18	0.07; 0.30	0.002
ANB (°)	0.34 (0.14)	0.39 (0.12)	0.05	0.01; 0.09	0.023
ANS-PNS/ Go-Gn (°)	0.92 (0.64)	1.10 (0.54)	0.18	0.02; 0.35	0.027
Occl/SN (°)	0.92 (0.67)	1.07 (0.52)	0.15	0.03; 0.26	0.016
SN/Go-Gn (°)	0.77 (0.45)	0.87 (0.36)	0.11	0.01; 0.21	0.033
Pog to NB (mm)	0.43 (0.21)	0.47 (0.17)	0.03	− 0.01; 0.07	0.150
N-S-Ba (°)	1.40 (0.98)	1.51 (0.72)	0.12	− 0.07; 0.30	0.216
**Dentoalveolar**					
Upper inc / ANS-PNS (°)	1.64 (0.96)	1.91 (0.81)	0.27	0.08; 0.47	0.008
Upper inc to NA (mm)	0.36 (0.23)	0.39 (0.14)	0.03	− 0.01; 0.08	0.129
Inter-incisal angle (°)	2.87 (2.04)	2.83 (1.42)	− 0.04	− 0.34; 0.26	0.793
Lower inc / GoGn (°)	2.33 (1.76)	2.32 (1.41)	− 0.01	− 0.20; 0.18	0.926
Lower inc to NB (mm)	0.38 (0.37)	0.38 (0.28)	0.01	− 0.03; 0.05	0.701

Variation is expressed as the standard deviation of 4 measurements of the SLC and 4 measurements of the RLC for each of the outcome variables. Differences in standard deviations of the SLC and the RLC were analyzed using a paired sample *t*-test. *SLC* = standard dose lateral cephalogram, *RLC* = reconstructed lateral cephalogram, 95%CI = 95% confidence intervals, # significance of results of paired sample *t*-test

Measurements on SLCs of SNA, ANS-PNS/Go-Gn, N-S/Ba, and Upper inc./ANS-PNS were significantly more often ≥ 2 (mm or degrees) than measurements on RLCs (Table 2).

**Table 2:** Number and percentage of observations with a difference ≥ 2.0 from the grand mean of linear and angular measurements performed on standard lateral cephalograms (*SLC*) and reconstructed lateral cephalogram (*RLC*)

Variable	Observations ≥ 2 mm or degrees from grand mean				
	SLC		RLC		*p*#
	N	%	N	%	
**Skeletal**					
SNA (°)	4	2	13	8	0.049
SNB (°)	3	2	5	3	0.727
ANB (°)	0	0	0	0	1.000
ANS-PNS/ Go-Gn (°)	5	3	21	12	0.002
Occl/SN (°)	7	4	7	4	1.000
SN/Go-Gn (°)	3	2	11	6	0.057
Pog to NB (mm)	0	0	0	0	1.000
N-S-Ba (°)	15	9	35	20	0.007
**Dentoalveolar**					
Upper inc / ANS-PNS (°)	15	9	44	26	< 0.001
Upper inc to NA (mm)	1	1	0	0	1.000
Inter-incisal angle (°)	34	20	37	22	0.813
Lower inc / GoGn (°)	22	13	31	18	0.272
Lower inc to NB (mm)	1	1	0	0	1.000

Grand mean is calculated for the 8 measurements per skull per outcome variable. The number of observations with a difference ≥ 2.0 mm or degrees from the grand mean was calculated per skull for each outcome variable and for each type of radiograph, # significance based on McNemar test. SLC and RLC measurements: 172 paired observations (4 × 43 SLC, 4 × 43 RLC)

### Reliability

#### Intra-observer reliability

For observer 1, the ICCs of the SLC measurements ranged from 0.95 to 0.99 and for the RLC from 0.88 to 0.98 (Table 3). The lower limit of the 95% confidence interval for the measurements on the SLC images ranged from 0.93 to 0.98 and for the RLC from 0.78 to 0.96.

**Table 3:** Intra-observer reliability of linear and angular measurements performed on standard lateral cephalograms (*SLC*) and reconstructed lateral cephalogram (*RLC*) of observer 1 and observer 2

Variable	**SLC**				**RLC**			
	Observer 1		Observer 2		Observer 1		Observer 2	
	ICC single measures	95% CI	ICC single measures	95% CI	ICC single measures	95% CI	ICC single measures	95% CI
**Skeletal**								
SNA (°)	0.98	0.97; 0.99	0.98	0.95; 0.99	0.93	0.90; 0.96	0.97	0.92; 0.99
SNB (°)	0.98	0.97; 0.99	0.98	0.94; 0.99	0.92	0.88; 0.95	0.97	0.92; 0.99
ANB (°)	0.98	0.97; 0.99	0.96	0.90; 0.99	0.98	0.96; 0.99	0.98	0.95; 0.99
ANS-PNS/Go-Gn (°)	0.98	0.96; 0.99	0.95	0.86; 0.98	0.95	0.91; 0.97	0.92	0.80; 0.98
Occl/SN (°)	0.96	0.93; 0.97	0.90	0.76; 0.97	0.93	0.88; 0.96	0.89	0.75; 0.97
SN/Go-Gn (°)	0.98	0.97; 0.99	0.95	0.88; 0.99	0.97	0.95; 0.98	0.95	0.86; 0.98
Pog to NB (mm)	0.95	0.93; 0.97	0.95	0.86; 0.98	0.95	0.92; 0.97	0.95	0.89; 0.99
N-S-Ba (°)	0.97	0.95; 0.98	0.65	0.36; 0.88	0.88	0.81; 0.93	0.88	0.74; 0.97
**Dentoalveolar**								
Upper inc/ANS-PNS (°)	0.97	0.96; 0.98	0.96	0.91; 0.99	0.96	0.94; 0.98	0.98	0.95; 0.99
Upper inc to NA (mm)	0.99	0.98; 0.99	0.98	0.95; 0.99	0.98	0.96; 0.99	0.99	0.97; 1.00
Inter-incisal angle (°)	0.98	0.96; 0.99	0.91	0.78; 0.97	0.94	0.91; 0.96	0.95	0.88; 0.99
Lower inc/GoGn (°)	0.95	0.93; 0.97	0.87	0.71; 0.96	0.86	0.78; 0.91	0.88	0.73; 0.96
Lower inc to NB (mm)	0.99	0.98; 0.99	0.98	0.95; 0.99	0.96	0.93; 0.98	0.98	0.95; 0.99

ICCs for observer 1 (RvB) are based on 344 observations (4 × 43 SLC, 4 × 43 RLC). ICCs for observer 2 (AD) are based on 80 observations (4 × 10 SLC, 4 × 10 RLC)

For observer 2, the ICCs of the SLC measurements ranged from 0.65 to 0.98 and for the RLC from 0.88 to 0.99 (Table 3). The lower limit of the 95% confidence interval for the measurements on the SLC images ranged from 0.36 to 0.95 and for the RLC from 0.73 to 0.95.

#### Inter-observer reliability

The ICCs of the measurements on SLCs ranged from 0.77 to 0.98 and the ICCs of the measurements on RLC ranged from 0.85 to 0.99 (Table 4). The lower limit of the 95% confidence interval for the measurements on the SLCs ranged from 0.58 to 0.95 and for the RLCs from 0.70 to 0.96.

**Table 4:** Inter-observer reliability of linear and angular measurements performed on standard lateral cephalograms (*SLC*) and reconstructed lateral cephalogram (*RLC*) (observers 1 and 2)

Variable	ICC observer 1 vs observer 2			
	SLC		RLC	
	ICC single measures	95% CI	ICC single measures	95% CI
**Skeletal**				
SNA (°)	0.98	0.95; 0.99	0.97	0.92; 0.99
SNB (°)	0.98	0.95; 0.99	0.97	0.92; 0.99
ANB (°)	0.97	0.94; 0.99	0.98	0.96; 0.99
ANS-PNS/ Go-Gn (°)	0.95	0.90; 0.99	0.94	0.86; 0.98
Occl/ SN (°)	0.89	0.77; 0.96	0.90	0.79; 0.97
SN/ Go-Gn (°)	0.96	0.90; 0.99	0.95	0.90; 0.99
Pog to NB (mm)	0.96	0.91; 0.99	0.97	0.92; 0.99
N-S-Ba (°)	0.77	0.58; 0.92	0.85	0.70; 0.95
**Dentoalveolar**				
Upper inc/ANS-PNS (°)	0.97	0.94; 0.99	0.98	0.95; 0.99
Upper inc to NA (mm)	0.98	0.95; 0.99	0.99	0.97; 1.00
Inter-incisal angle (°)	0.94	0.87; 0.98	0.96	0.90; 0.99
Lower inc / GoGn (°)	0.91	0.81; 0.97	0.91	0.80; 0.97
Lower inc to NB (mm)	0.98	0.96; 0.99	0.98	0.96; 0.99

ICCs are based on 160 observations 2 × (4 × 10 SLC, 4 × 10 RLC)

## Discussion

In the present study, we analyzed the differences in variation in measurement results performed on SLC and RLC. We compared standard deviations of measurements performed on SLC and RLC and the number of observations falling outside the range of 2 mm/degrees from the grand mean. Furthermore, we assessed intra- and interobserver reliability. In order to use RLC for orthodontic purposes, the cephalometric measurements on the images must meet a clinically acceptable degree of variation and reliability.

To the best of our knowledge, only one feasibility study (*n* = 4) [21] has been published that investigated a similar question: What is the quality of (simulated) lower dose images extracted from standard dose CBCT? The aim of that two-part study was to analyze landmark identification as well as the diagnostic value of images obtained using an ultra-low-dose reduced projection (sparse) views algorithm applied to existing CBCT data. The number of projection views is in direct proportion with the lowering of radiation dose. Assessment of diagnostic quality was studied by evaluating radiographs of various projection views on a visual analog scale by different dental specialists. Remarkably, that study found no statistically significant differences in the quality of images at 25% projection views as compared to 100% projection views. Assessment of 2D landmark identification derived from CBCT data at different projection views was also conducted. Due to the small sample size of the second part of that study, inter- and intra-observer reliability and accuracy testing were not conducted. Therefore, comparisons with our results are not possible.

When comparing two 2-dimensional imaging modes of a 3-dimensional object, like a skull, a problem is the lack of a gold standard. Measurements in the midsagittal plane cannot be performed on an intact dry skull to validate them. Furthermore, it is unknown which type of lateral cephalogram leads to more consistent measurements. For this reason, it was decided to analyze differences in variation in measurements on the two imaging modes (SLC and RLC) and with respect to a grand mean. Observations within the range of 2.0 mm or degrees were considered clinically acceptable. This criterion is an arbitrarily chosen one but is a generally accepted value in most other studies at this point [12, 18–20].

Although mean SDs for 7 out of 13 variables were significantly smaller for SLCs than for RLCs (Table 1), mean SDs and 95% CI for both types of images of these variables are very small (< 2 mm/degrees) and it is questionable whether this difference in variation of measurement results is clinically relevant. Mean SDs of the measurements of inter-incisal angle and lower-incisor to GoGn angle were larger than 2 mm/degrees for SLC and RLC but the clinical implications are the same for both image modalities. Determining lower incisor apex and Gonion on SLC in general is the least reliable of all cephalometric landmarks [22]. Although measurements on RLCs were more often outside the range of 2 mm/degrees than measurements on SLC (Table 2), in only 4 of the 13 variables, the measurements on RLCs were significantly more often outside the range.

The intra-observer reliability of the first observer was very good. The lower border of the 95% CI of the ICC was above 0.90 for all variables on SLC and in 9 out of 13 variables on RLC. The intra-observer reliability of the second observer was slightly lower and 95% CIs were a bit wider but were based on observations on 10 skulls. Still, the lower border of the 95% CI of the ICC was above 0.90 in six out of 13 variables on SLC and in six out of 13 variables on RLC. Measurements of N-S-Ba of the second observer were more consistent on RLC than on SLC, while measurements of this angle by the first observer were more consistent on SLC than on RLC. It is even more remarkable because measurements of N-S-Ba on RLC of observer 1 were significantly more often outside the range of 2 mm or degrees than measurements on SLC. We have no plausible explanation for this phenomenon.

Inter-observer reliability was good too. The lower border of the 95% CI of the ICC was above 0.90 in 9 out of 13 variables on SLC and on RLC. Reliability of measurements of N-S-Ba was the lowest, but they were better on RLC (ICC = 0.85) than on SLC (ICC = 0.77) although the difference is small. The reason could be coincidental individual observer errors.

The routine need of a lateral cephalogram for orthodontic diagnosis and treatment planning has been questioned because the availability of a cephalometric radiograph and analysis did not influence treatment decisions in adolescents with a class II division 1 malocclusion [23–25]. The diagnostic added value of CBCTs besides the traditional PAN and LC for orthodontic purposes is not yet clear and so far there is only evidence for its effectiveness in the diagnosis of impacted canines [1, 3, 5–7, 23]. On the other hand, as stated in the “Introduction” section of this paper, CBCTs in general contain more information with less over-projection than a single PAN, so visibility of structures is better on a CBCT than on a conventional PAN [2].

Considering the abovementioned small differences in variation of measurements on RLC compared to SLC, we could accept this in exchange for a lower radiation exposure per patient and the added value of three-dimensional information. As pointed out in the “Introduction” section of this paper, the combination of the traditional PAN and SLC (27–30 μSv) results in a larger radiation dose than a single ULD LD CBCT (11–18 μSv) [8, 9, 14, 15, 17]. When in every new orthodontic patient exam the conventional PAN and SLC are replaced by one ULD-LD CBCT, this would result in a radiation reduction of 9–19 μSv per patient. We would like to stress that this does not hold true for replacement of a conventional PAN and SLC by one normal dose CBCT [26], which would result in a 5–sevenfold dose increase as already stated in the “Introduction” section of this paper [8, 9].

It is the clinician’s obligation to reduce radiation as much as possible, and to decide in which individual treatment situation an increase in radiation exposure is justified. Since the quality of filters and setting options are subject to continuous improvement [21], it is obvious that more research will be needed to optimize the image quality of ULD-LD CBCT reconstructed lateral cephalograms.

## Limitations

A limitation of this research is that images of dry skulls were used in which the soft tissues were simulated. As a result, a comparative study of measurements on soft tissue landmarks could not be conducted. Although it has been shown that an EPS box with 2-cm-thick walls covered with a 1-cm-thick layer of utility wax is effective in simulating soft tissue in most regions, the difference between the two types of images with real soft tissues could not be determined. Conducting this type of research in patients is ethically questionable. Another option would have been using cadaver heads, which would have given a better representation of reality. The reason why we did not choose cadaver heads was that we could not obtain enough cadaver heads of adolescents and adults with a complete dentition. If we had used cadaver heads, we would not have been able to obtain such a large number of skulls (*N* = 43), which would have reduced statistical power.

## Conclusions

Based on the lower radiation dose and the small differences in variation in cephalometric measurements on reconstructed LC compared to standard dose LC, ULD-LD CBCT with reconstructed LC should be considered for orthodontic diagnostic purposes.

## Supplementary Information

Below is the link to the electronic supplementary material.

Supplementary file1 (DOCX 17 KB)
