# Supplementary material for: Precision of orthodontic cephalometric measurements on ultra low dose-low dose CBCT reconstructed cephalograms
Source: Clin Oral Investig. 2021 Aug 28;26(2):1543–50. doi: 10.1007/s00784-021-04127-9 (PMC8816531; doi:10.1007/s00784-021-04127-9)
Supplement: Supplementary file 1 — Supplementary file1 (DOCX 17 KB) [file 784_2021_4127_MOESM1_ESM.docx]

**Supplementary table 1**

Skeletal and dental cephalometric landmarks used in this study.

| **Skeletal landmarks** | |
| --- | --- |
| Po | Porion |
| Or | Oribitale |
| S | Sella turcica |
| N | Nasion |
| ANS | Anterior Nasal Spine |
| PNS | Posterior Nasal Spine |
| A | A-point |
| B | B-point |
| Po | Pogonion |
| Gn | Gnathion |
| Go | Gonion |
| Ba | Basion |
| Ar | Articulare |
|  |  |
| **Dental landmarks** | |
| U1i | Upper Incisor tip |
| U1r | Upper Incisor root apex |
| L1i | Lower Incisor tip |
| L1r | Lower Incisor root apex |
| II | Interincisal point |
| U6 MB cusp | Upper first molar mesiobuccal cusp |
| L6 MB cusp | Lower first molar mesiobuccal cusp |

**Supplementary table 2**

Skeletal and dentoalveolar cephalometric variables (angles and distances) used in this study.

| **Variable** |  |  |  |  |
| --- | --- | --- | --- | --- |
| **Skeletal** |  |  |  |  |
| SNA (^0^) | Angle between line SN and line NA | | |  |
| SNB (^0^) | Angle between line SN and line NB | | |  |
| ANB (^0^) | Angle between line AN and line NB | | |  |
| ANS-PNS-/-Go-Gn (^0^) | Angle between line ANS - PNS and line Go - Gn | | |  |
| Occl-/ -SN (^0^) | Angle between occlusal plane (U6-II) to line SN | | |  |
| SN-/-Go-Gn (^0^) | Angle between line SN and line Go - Gn | | |  |
| Pog to NB (mm) | Distance between Pog perpendicular to NB | | |  |
| N-S-Ba (^0^) | Angle between line NS and line Ba-S | | |  |
| **Dentoalveolar** |  |  |  |  |
| Upper inc-/-ANS-PNS (^0^) | Angle between Upper incisor and line ANS-PNS | | | |
| Upper inc to NA (mm) | Distance between Upper incisor and line NA | | |  |
| Inter-incisal angle (^0^) | Angle between Upper incisor and Lower incisor | | |  |
| Lower inc-/- GoGn (^0^) | Angle between Lower incisor and line Go - Gn | | |  |
| Lower inc to NB (mm) | Distance between Lower incisor perpendicular to line NB | | | |
